# Unveiling an ALS Blood Transcriptomic Signature: A Machine Learning Classifier Distinct from Neurodegenerative Controls

**DOI:** 10.1007/s12021-026-09780-7

**Published:** 2026-04-29

**Authors:** Elisa Gascón, Ana Cristina Calvo, Pilar Zaragoza, Rosario Osta

**Affiliations:** 1 LAGENBIO, Faculty of Veterinary, University of Zaragoza, Miguel Servet 177, 50013 Zaragoza, Spain; 2 Centre for Biomedical Research, Neurodegenerative Diseases (CIBERNED), Av. Monforte de Lemos 3-5, 28029 Madrid, Spain; 3 Agroalimentary Institute of Aragon (IA2), University of Zaragoza, 50013 Zaragoza, Spain; 4 Institute of Health Research of Aragon (IISA), Av. San Juan Bosco 13, 50009 Zaragoza, Spain

**Keywords:** Amyotrophic lateral sclerosis, Transcriptomics, Machine learning, Biomarkers, Neuroinflammation, Peripheral blood, Neurodegenerative diseases

## Abstract

The absence of accessible and reliable biomarkers constitutes a critical barrier for the early diagnosis and stratification of neurodegenerative diseases. While peripheral blood offers a minimally invasive window into systemic pathophysiology, identifying molecular signatures that survive biological heterogeneity and technical noise remains an unresolved challenge. In this study, this issue was addressed through a comparative systemic transcriptomic analysis of Amyotrophic Lateral Sclerosis (ALS), Alzheimer’s disease (AD), and Parkinson’s disease (PD) in whole blood, implementing a comprehensive workflow integrating unsupervised network analysis and supervised machine-learning methods. By employing LASSO regression and cross-validation across independent external cohorts, a stable and specific transcriptomic signature for ALS was identified, comprising key crosstalk genes involved in systemic immune dysregulation and microglial function, including *CTSS, PTEN, IL18, PTPRC*, and *CSF1R*. In contrast, AD and PD exhibited weak transcriptomic signatures with poor predictive reproducibility, suggesting a distinctive systemic pathology in ALS. In addition, the study confirms the superiority of linear modeling for this genomic signature: while complex non-linear algorithms, specifically Radial Basis Function (RBF) kernel Support Vector Machine (SVM) and Random Forest, displayed high initial performance, they collapsed due to overfitting during external validation. Conversely, the linear LASSO model demonstrated superior robustness and generalizability (AUC 0.74). In conclusion, this study not only defines a unique systemic immunotranscriptomic signature for ALS, distinguishable from other neurodegenerative pathologies, but also establishes interpretability and linear simplicity as essential factors for developing reproducible blood-based biomarkers with clinical translational potential.

**Graphical Abstract:**

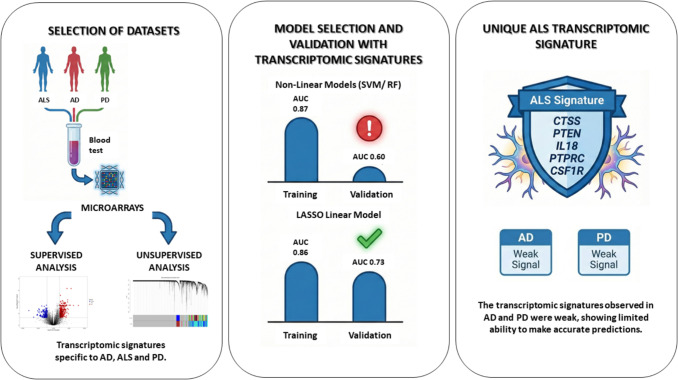

**Supplementary Information:**

The online version contains supplementary material available at 10.1007/s12021-026-09780-7.

## Highlights

Identification of a robust and specific transcriptomic signature in blood for ALS versus AD and PD using supervised and unsupervised methods.

The linear LASSO model outperforms SVM and Random Forest in generalisation and robustness.

The signature validates a systemic pattern of immune and microglial dysregulation.

External validation confirms the stability of the ELA transcriptome signature compared to the EA/EP transcriptome signatures.

Proposal for a set of accessible and interpretable blood biomarkers for ALS with high clinical potential.

**Supplementary Information:**

The online version contains supplementary material available at 10.1007/s12021-026-09780-7.

## Introduction

Neurodegenerative diseases represent one of the foremost healthcare and socioeconomic challenges of the twenty-first century. These are progressive and debilitating disorders for which no cure exists, they predominantly affect older adults, the age of onset can vary significantly depending on the specific disease and genetic factors. They are primarily characterized by neuronal loss and the progressive degeneration of specific tissues within the nervous system. This group includes prevalent pathologies such as Alzheimer’s disease (AD) and Parkinson’s disease (PD), while others, such as Amyotrophic Lateral Sclerosis (ALS), are considered rare diseases (Temple, 2023).

Currently, although there is no definitive cure for these diseases, there are effective FDA-approved therapies that can improve survival rates and/or reduce the burden of symptoms. Approved medications include Memantine, Donepezil, Rivastigmine, and Galantamine for Alzheimer’s disease; non-steroidal anti-inflammatory drugs, dopaminergic agonists, and monoamine oxidase B inhibitors, among others, to alleviate the effects of Parkinson’s disease (Fołta et al., 2025); and finally, Edaravone, Riluzol and Tofersen as the only approved drugs for the treatment of Amyotrophic Lateral Sclerosis (Gascón et al., 2024a, 2024b; Hamad et al., 2025). However, early and accurate diagnosis remains a fundamental clinical obstacle because clinical diagnosis relies on symptoms that manifest late in the disease progression, when neurodegeneration is already at an advanced stage, which limits the efficacy of any therapeutic strategy and the proper stratification of patients.

Each of these diseases exhibits distinct features in relation to their etiology: the accumulation of β-amyloid plaques and neurofibrillary tangles of hyperphosphorylated tau in AD (Cai et al., 2022; Gascón et al., 2024a, 2024b); loss of dopaminergic neurons in the substantia nigra and the presence of protein inclusions known as Lewy bodies in PD (Tansey et al., 2022); and progressive degeneration of upper and lower motor neurons in the spinal cord and the brainstem in ALS (Maity & Kaundal, 2025). Although these three diseases present with distinct clinical and histopathological differences, at the molecular level, they share convergent mechanisms such as protein aggregation, neurotoxicity, and notably, a dysregulation of the innate immune response that manifests as chronic neuroinflammation (Shi & Yong, 2025; Wilson et al., 2023).

The convergence of molecular mechanisms among these diseases offers a unique opportunity for the development of biomarkers. Because access to CNS tissue is a barrier to early diagnosis, we hypothesize that this shared pathology may be reflected in the blood. Therefore, the objective of this study is to investigate a common genetic background in this accessible tissue, using it as a molecular and functional basis to construct disease-specific biomarker panels and to evaluate their ability to discriminate between affected individuals and healthy controls using predictive classification models.

## Methods

### Selection of Datasets

A comprehensive search was conducted in the Gene Expression Omnibus (GEO) database using the keywords ‘ALS,’ ‘Alzheimer,’ and ‘Parkinson.’ Several stringent minimum criteria were established for dataset selection: (i) both patient and control groups had to comprise over 100 samples; (ii) all samples within a given dataset were required to originate from the same tissue type; (iii), consistent analytical platforms across samples were mandatory. Sample size was determined by the availability of high-quality public whole-blood datasets meeting our inclusion criteria (*N* > 100) to ensure statistical robustness.

Adhering to these criteria, the following datasets were selected (Table 1):

**Table 1:** Selection of datasets for the present study

GEO	Disease	Patient Samples	Control samples	Platform	Tissue
GSE112676	ALS	233	508	Illumina HumanHT-12 V3.0 expression beadchip	Whole blood
GSE99039	PD	205	233	Affymetrix Human Genome U133 Plus 2.0 Array	Whole blood
GSE140829	AD	204	249	Illumina HumanHT-12 v4 Expression BeadChip	Whole blood

To validate the obtained results, an independent cohort, distinct from the one used in the original study, was required. The previously defined selection criteria were applied to each disease, with the exception of PD. In this specific instance, due to the scarcity of cohorts containing over 100 whole blood samples, three separate datasets were chosen and subsequently integrated into a single validation set, applying batch correction using ComBat (see Fig. 4 in the Supplementary Material) (Johnson et al., 2007). The datasets used for validation are presented in Table 2.

**Table 2:** Selection of datasets for validation in the present study

GEO	Disease	Patient Samples	Control samples	Platform	Tissue
GSE112680	ALS	164	137	Illumina HumanHT-12 V3.0 expression beadchip	Whole blood
GSE57475	PD	93	49	Illumina HumanHT-12 V3.0 expression beadchip	Whole blood
GSE72267	PD	40	20	Affymetrix Human Genome U133A 2.0	Whole blood
GSE18838	PD	18	12	Affymetrix Human Exon 1.0 ST Array	Whole blood
GSE63061	AD	139	134	Illumina HumanHT-12 v4 Expression BeadChip	Whole blood

Information on demographic data, such as age and sex, is available in Table 1 of the Supplementary Material.

### Differential Expression Analysis

For the differential expression analysis between healthy and affected individuals, each disease cohort was processed and analyzed independently following the methodology described by Gascón et al. (Gascón et al., 2024a, 2024b). Briefly, raw data were normalized using the RMA method, and differential expression was determined using the limma package. To account for the inherent technical variability and distinct data distributions across the microarray platforms, we applied cohort-specific log2 fold-change (log2-FC) thresholds. This calibration was necessary because these platforms possess distinct dynamic ranges and baseline noise levels, which affect the observed magnitude of differential expression. For Amyotrophic Lateral Sclerosis, a significant differential expression was defined as log2 FC > 0.2 (1.15-fold change). For both Alzheimer’s disease and Parkinson’s disease, the threshold was set at log2 FC > 0.1 (1.07-fold change). Across all cohorts, statistical significance was set at an FDR-adjusted *p*-value < 0.05.

### Overlapping DEGs

To identify overlapping DEGs across the three diseases, the R package VennDiagram (v1.7.3) (Chen & Boutros, 2011) was utilized to generate a Venn diagram. For this purpose, three distinct DEG sets were created, one for each disease. Each set included both up-regulated and down-regulated genes. We specifically identified four combinations of overlapping genes: 1) genes overlapping across AD, PD, and ALS; 2) genes overlapping between AD and PD; 3) genes overlapping between ALS and AD; and 4) genes overlapping between ALS and PD.

### Protein–Protein Interaction (PPI) Network Construction and Identification of Hub Genes

To identify hub genes, STRING database (https://string-db.org/, acessed on 9 May 2025) was used for predict and analyze protein–protein interactions (PPIs) among genes overlapping across the different conditions. From these four combinations, four PPI networks were generated using a medium confidence score of 0.400. These networks were then visualized using Cytoscape (v3.9.1) (Shannon et al., 2003). Topological analysis of each network were performed using the cytoHubba plugin (Chin et al., 2014). Among the 11 methods available in this plugin, four established methods were selected: Maximal Clique Centrality (MCC), Degree Centrality, Edge Percolated Component (EPC), and Maximum Neighborhood Component (MNC). In this study, these four methods were applied to identify the top 20 genes exhibiting the highest values for each respective method. Finally, genes that overlapped across the results of all four methods were considered Hub genes for each group.

### Weighted Gene Co-Expression Network Analysis (WGCNA)

Gene co-expression network analysis was performed independently for each disease using the WGCNA R package (version 1.73) (Langfelder & Horvath, 2008). Optimal values for the weighted parameters of the adjacency functions were determined using the *pickSoftThreshold* function, testing soft thresholds ranging from 1 to 20. An appropriate was identified for the subsequent network construction for each disease to ensure that platform-specific architectures were respected before any cross-disease comparison. Subsequently, a weighted adjacency matrix was constructed, and hierarchical clustering based on the topological overlap matrix (TOM), with a dissimilarity measure (1-TOM), was used to group genes. This hierarchical clustering approach enabled the identification of distinct modules of highly interconnected genes. These identified subnetworks, or co-expression modules, represent groups of tightly linked genes that are likely to be key players contributing to relevant biological pathways. Finally, the correlation of each module with disease status was calculated, and modules exhibiting a significant correlation threshold greater than 0.2 were selected for further analysis.

### Enrichment and Functional Analysis

Functional enrichment analysis was performed using the Database for Annotation, Visualization, and Integrated Discovery (DAVID; https://davidbioinformatics.nih.gov/ acessed on 9 June 2025). Specifically, the analysis focused on Gene Ontology (GO) terms, which were categorized into three domains: (1) biological processes, (2) molecular functions, and (3) cellular components. Statistical significance was determined using a stringent criterion: GO terms with a *p*-value < 0.05 were considered statistically significant.

### Identification and Validation of Key Crosstalk Genes

Key crosstalk genes were defined as the intersection between the Hub genes identified from the protein–protein interaction (PPI) networks (derived from DEGs for each disease, as described in Sect. “Differential Expression Analysis”) and the key module genes obtained by the WGCNA (described in Sect. “Weighted Gene Co-Expression Network Analysis (WGCNA)”). Each of the four possible combinations involving the three diseases (AD-ALS-PD, ALS-AD, PD-AD, and ALS-PD) was analyzed. Importantly, we did not select only genes that had 100% overlap in all Hub genes and all modules for each combination. Instead, we included all genes demonstrating an overlap between the Hub genes and one or more specific modules within each combination.

The mRNA expression levels (represented as normalized expression values derived from the microarray datasets) of these identified crosstalk genes were compared in case and control groups. Prior to statistical testing, extreme expression outliers were filtered out using the Interquartile Range (IQR) method. The assumption of equal variances was evaluated using Levene’s test. Subsequently, an independent Student’s *t*-test was applied for genes with equal variances, whereas Welch’s *t*-test was utilized for genes violating this assumption. An FDR-adjusted *p*-value less than 0.05 was considered statistically significant. The relative expression levels of the crosstalk genes in both case and control groups were visualized using box plots generated with the R package ggplot2 (Wilkinson, 2011).

### Construction of a Classification Model

To evaluate the predictive power of the identified key crosstalk genes (17 genes for AD, 20 for ALS, and 15 for PD) and to select the most relevant genes, Least Absolute Shrinkage and Selection Operator (LASSO) logistic regression analysis was applied (Tibshirani, 1996). This method performs simultaneous variable selection and L1 regularization, reducing the coefficients of less important features to zero and retaining only the most relevant predictive genes, thereby producing a sparse model. The strength of the penalization term in LASSO regression is controlled by a tuning parameter, λ. When *λ* = 0, no coefficients (features) are removed. As the value of λ increases, more coefficients are set to zero and consequently eliminated.

The LASSO model was developed utilizing the scikit-learn (sklearn) Python library (Pedregosa et al., 2011). To further validate the performance of the linear LASSO model, it was compared with two non-linear algorithms: Support Vector Machine (SVM) with a radial basis function (RBF) kernel (Noble, 2006) and Random Forest (RF) (Breiman, 2001). These models were evaluated to determine if non-linear relationships (via SVM) or complex feature interactions (via RF) within the gene signature provided superior predictive power. A tenfold stratified cross-validation was applied to evaluate model performance and select optimal hyperparameters (detailed in Table S2 of the Supplementary Material).

The predictive quality of the classification model was assessed by calculating the number of true positives (TP), false negatives (FN), true negatives (TN), and false positives (FP). Based on these criteria, several metrics were derived: Accuracy, Precision, Recall, Specificity, F1 Score, the ROC curve and Confusion Matrix (Table 3).

**Table 3:** Confusion Matrix

	Actual values	
Predicted values	True positive (TP)	False positive (FP)
	False negative (FN)	True negative (TN)

$$Accuracy: \frac{TP+TN}{\left(TP+TN+FP+FN\right)}$$

$$Precision: \frac{TP}{\left(TP+FP\right)}$$

$$Recall: \frac{TP}{\left(TP+FN\right)}$$

$$Specifity: \frac{TN}{\left(TN+FP\right)}$$

$$F1 Score: \frac{TP}{TP+\frac{1}{2}\left(FP+FN\right)}$$

### Validation of Key Crosstalk Genes and Classification Model in Independent External Cohorts

To increase the confidence in our findings, we validated the expression of the identified crosstalk genes and the performance of the three classification models (LASSO, SVM and Random Forest) in several independent external cohorts, distinct from our original dataset. These validation cohorts are detailed in the preceding “Selection of datasets” section. We performed a comparative analysis of the models on these cohorts, adhering to the methodology previously described.

### Study Design

**Fig. 1:**
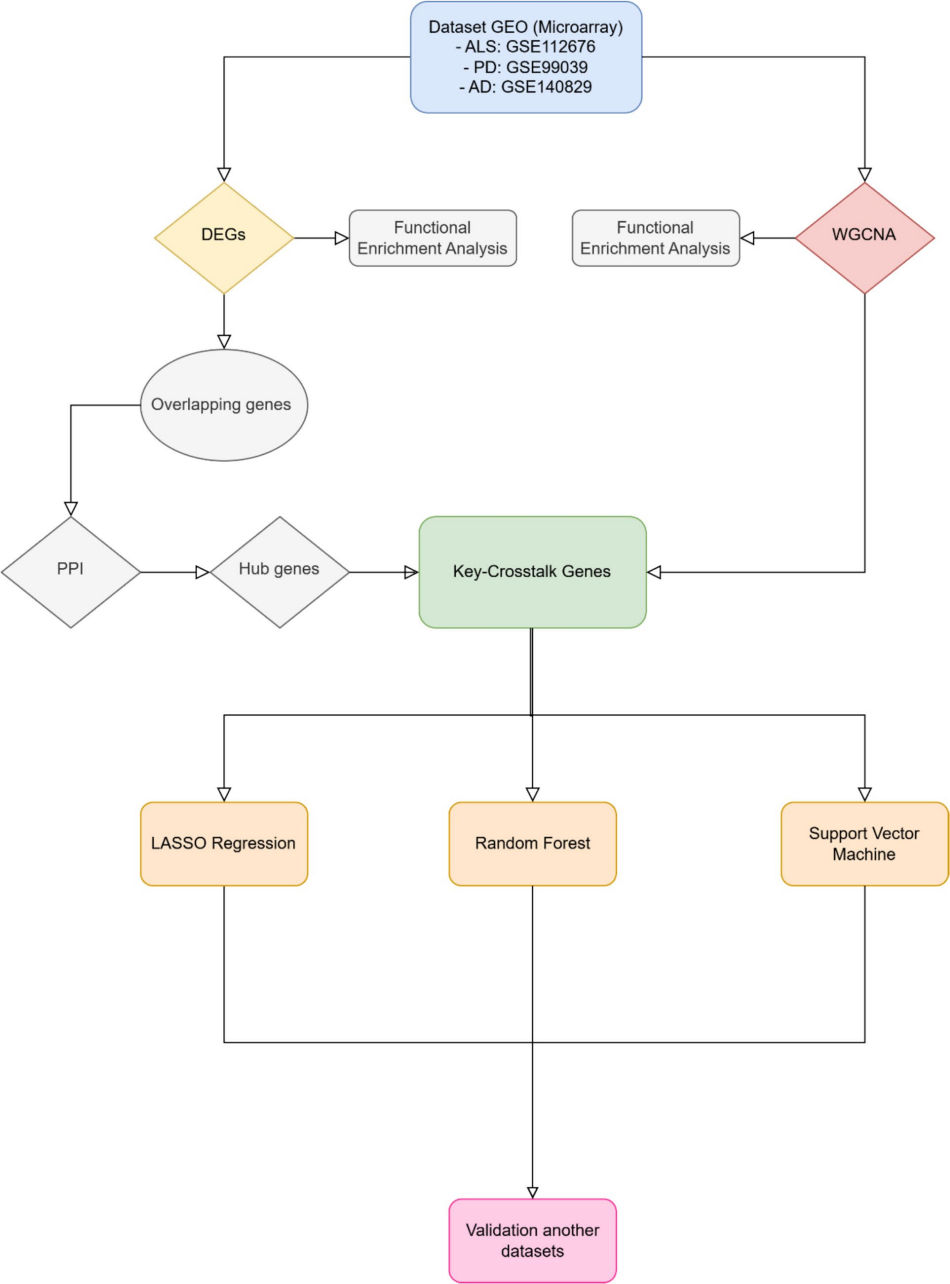
The study flowchart of the current research

Figure 1 illustrates the experimental workflow designed for this study. First, three distinct datasets, one for each disease (ALS, AD, and PD), were selected. Subsequently, both supervised (Differential Expression Analysis) and unsupervised (Weighted Gene Co-expression Network Analysis (WGCNA)) analyses were performed on each dataset. From the differentially expressed genes (DEGs), those common to all three diseases were identified. These common DEGs were then intersected with the key module genes identified through WGCNA. The resulting genes were designated as key crosstalk genes. Finally, their classification potential was analyzed using LASSO regression. To validate these key crosstalk genes, their behavior was re-evaluated by performing differential expression analysis and LASSO analysis on an independent cohort to confirm the reproducibility of their observed patterns.

## Results

### Differential Expression Analysis and Identification of Hub Genes

Our differential expression analysis revealed distinct gene profiles across the three diseases. For AD, we identified 353 upregulated genes and 191 downregulated genes. In ALS, 881 genes were found to be upregulated and 592 downregulated. Finally, for PD, 1054 genes were upregulated and 223 downregulated. Their corresponding volcano-plot graphs are shown in Fig. 1 of the Supplementary Material.

Subsequently, we conducted a functional enrichment analysis for both the upregulated and downregulated genes within each disease (Table 3 of the Supplementary Material). For AD, the most enriched biological processes among upregulated genes included signal transduction, innate immune response, protein phosphorylation, actin filament organization, and cellular response to lipopolysaccharides. On the other hand, downregulated genes in AD were enriched in biological processes such as apoptosis, ribosomal subunit biogenesis, protein translation, cell cycle regulation, T-cell receptor signaling, and calcium-mediated signaling. In ALS, upregulated genes were strongly associated with biological processes such as apoptosis, inflammatory response, signal transduction, positive regulation of RNA and DNA transcription, and protein ubiquitination. Conversely, downregulated genes in ALS were implicated in protein folding, phosphorylation, immune cell regulation (T-cells), and calcium-mediated signaling. Finally, in PD, upregulated genes were associated with biological processes such as regulation of transcription by RNA polymerase II, signal transduction, apoptosis, phosphorylation, and inflammatory response. Meanwhile, downregulated genes in PD were involved in negative regulation of cell proliferation, cell surface receptor signaling pathways, signal transduction, and angiogenesis.

**Fig. 2:**
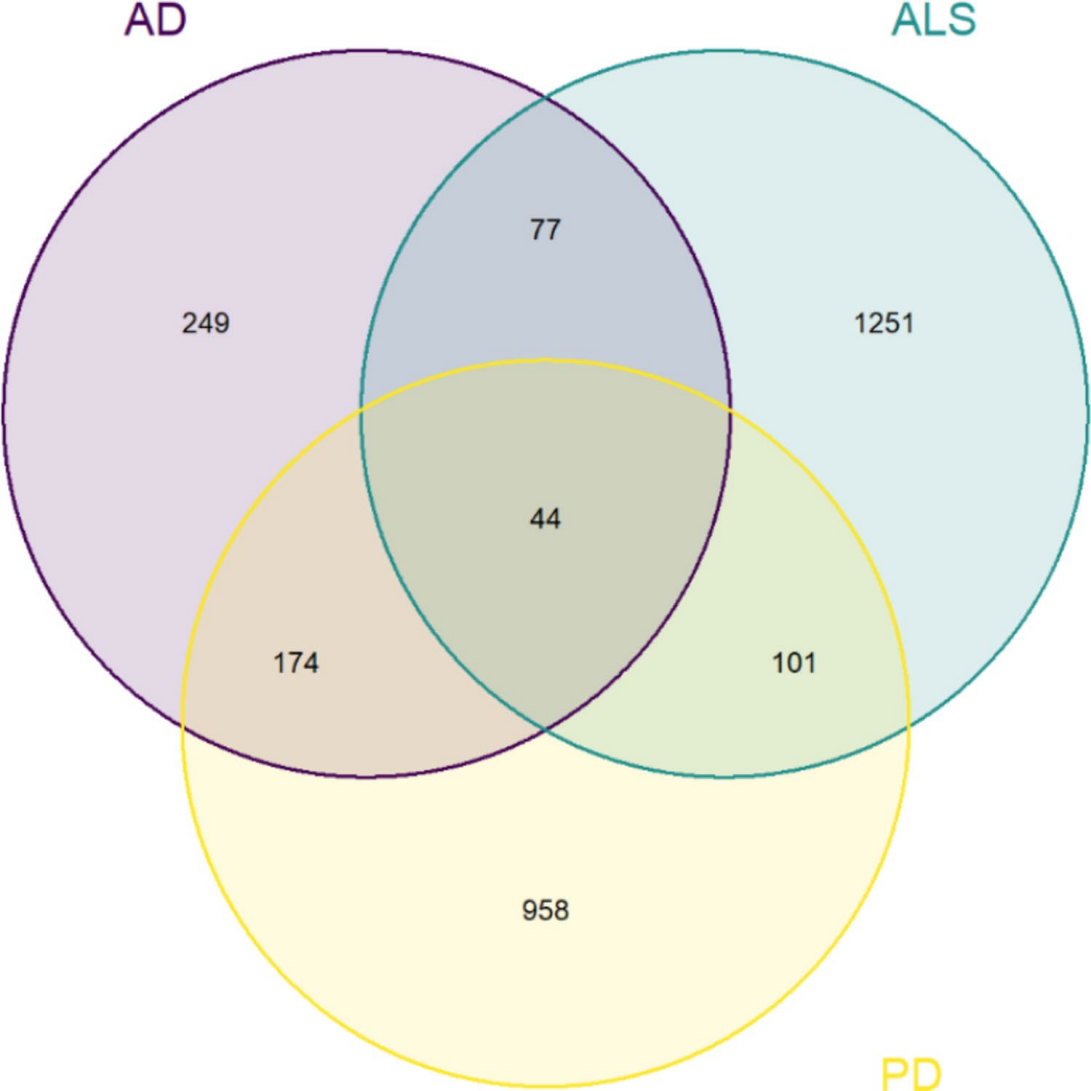
Venn diagrams for AD, ALS and PD suggested 44 target genes in common among these three diseases, while 249 genes were specifically identified in AD, 1251 in ALS and 958 in PD

After identifying the differentially expressed genes (DEGs), both upregulated and downregulated, for each disease, we used a Venn diagram (Fig. 2) to visualize overlapping gene sets. This allowed us to pinpoint common genes across the three diseases and their various combinations based on statistical significance, regardless of their direction of effect (up- or down-regulation) in each disease. Specifically, we found 44 genes overlapping among AD, PD, and ALS; 174 genes common to AD and PD; 77 genes shared by AD and ALS; and 101 genes overlapping between ALS and PD.

Finally, following the construction of protein–protein interaction (PPI) networks and subsequent clustering, the Hub Genes were identified and are presented in the Table 4:

**Table 4:** Hub Genes identified for each combination of the three diseases

Combination	Number of hub genes	Names of hub genes
AD-ALS-PD	7	*MMP9, C5AR1, TLN1, ITGAM, LILRB2, PGLYRP1, ARG1*
AD-PD	15	*NCF4, VAV1, CORO1A, HCK, NCF1, PTPN6, MYO1F, MSN, HCLS1, PFN1, WAS, FGR, CSF3R, MAPK1, SPI1*
AD-ALS	15	*URI1, NKTR, RGS19, OPTN, TPT1, RPL22, RBM25, ZRANB2, FOS, BIRC3, RPS6KB1, RPS29, RPL15, EIF4A2, TAX1BP1*
ALS-PD	13	*PRF1, LILRB1, IL18, CSF1R, IRF4, TLR4, GZMB, PTEN, TNFSF13B, PTPRC, GNLY, CTSS, ERBB2*

### Identification of Weighted Gene Co-Expression Network Analysis (WGCNA) Modules and Key Crosstalk Genes

**Fig. 3:**
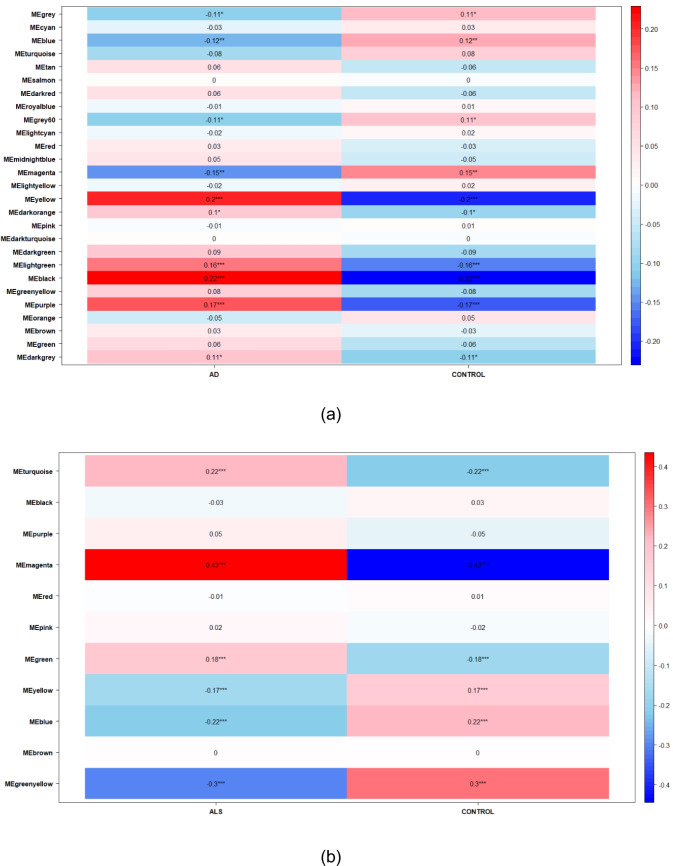

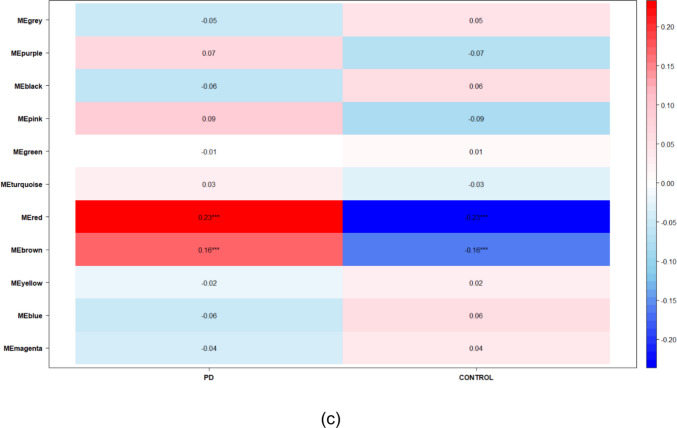
Representation of the correlation matrix of the WGCNA modules for each disease: **a** Alzheimer’s disease, **b** Amyotrophic Lateral Sclerosis and **c** Parkinson’s disease

For the WGCNA, we selected modules with a significance greater than 0.2. In the case of AD, the chosen modules were MEblack (186 genes) and MEyellow (420 genes) (Fig. 3.a). For ALS, several modules were identified: MEblue (1489 genes), MEgreenyellow (28 genes), MEmagenta (42 genes), and MEturquoise (2255 genes) (Fig. 3.b). Finally, for PD, a single module, MEred, comprising 171 genes, was found (Fig. 3.c).

Once all modules corresponding to each disease were identified, an ontological enrichment analysis was performed to ascertain the most relevant processes (Table 4 of the Supplementary Material). For AD, prominent biological processes included signal transduction, innate immune and inflammatory response, canonical NF-kappaB signal transduction, vesicle-mediated transport, and actin cytoskeleton and filament organization, among others. Conversely, for ALS, some of the key biological processes observed were apoptotic processes, phosphorylation, protein ubiquitination, and regulation of DNA-templated transcription. Finally, for PD, the most relevant biological processes were those related to inflammatory response and innate immunity. Overall, the identified processes showed similarities to those obtained from the differentially expressed genes of each respective disease.

To identify the key crosstalk genes, we intersect the Hub Genes with one or more specific modules derived from the WGCNA analysis within each of the four disease combinations. Our aim was to detect not only genes that showed a 100% overlap between these categories but also key genes that coincided with at least one module from the WGCNA analysis. A total of 24 unique key crosstalk genes were obtained. From the AD-ALS-PD combination, we identified four genes: *LILRB2*, *TLN1*, *ITGAM*, and *C5AR1*. For the AD-ALS combination, nine genes were obtained: *RPL15*, *FOD*, *EIF4A2*, *NKTR*, *TPT1*, *RBM25*, *RPL22*, *TAX1BP1*, and *RPS6KB1*. For the ALS-PD combination, seven genes were obtained: *IL18*, *PTPRC*, *PTEN*, *CTSS*, *TNFSF13B*, *TLR4*, and *CSF1R*. Finally, the AD-PD combination resulted in four genes: *NCF4*, *MYO1F*, *VAV1*, and *HCLS*.

**Fig. 4:**
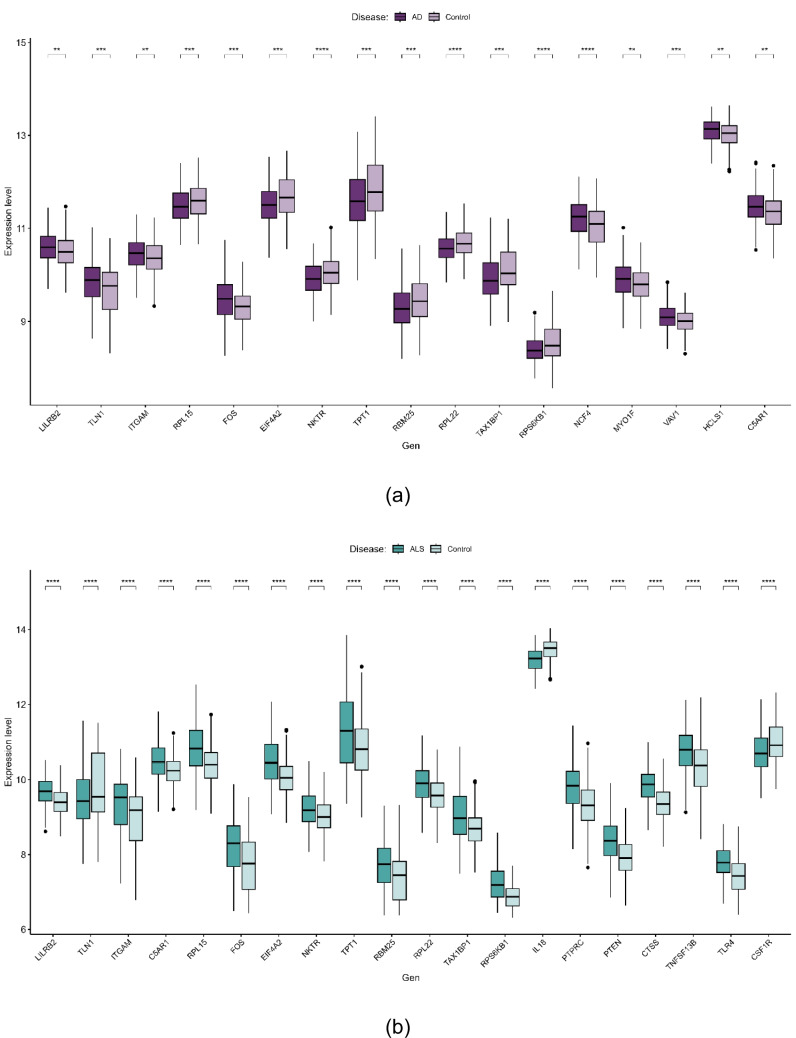

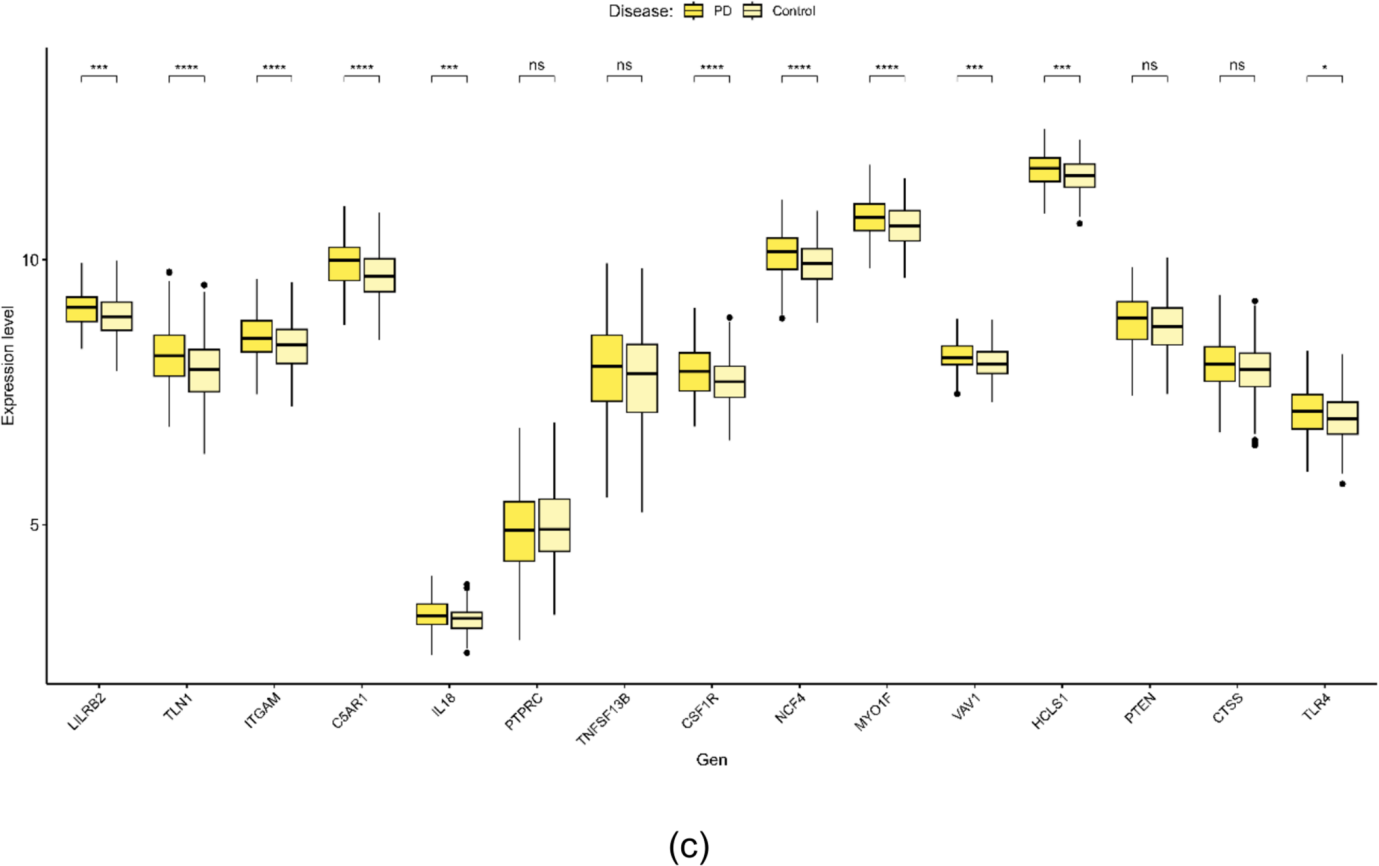
mRNA expression levels of key crosstalk genes in each neurodegenerative disease. Boxplots display the distribution of normalized expression values for each gene in Alzheimer’s disease (AD) (**a**), Amyotrophic Lateral Sclerosis (ALS) (**b**), and Parkinson’s disease (PD) (**c**). Statistical significance between patient and control groups was determined using either an independent Student’s *t*-test or Welch’s *t*-test, conditional on the assumption of equal variances as evaluated by Levene’s test

To evaluate the mRNA expression levels of each identified gene, statistical comparisons between case and control groups within their respective disease cohorts were performed using either Student’s or Welch’s *t*-tests, depending on variance equality. The results are shown in Fig. 4 and Table 5 of the Supplementary Material.

As shown in Fig. 4, all genes exhibited statistical significance in their respective cohorts, with the exception of four genes (*PTPRC*, *TNFSF13B*, *PTEN*, and *CTSS*) in the Parkinson’s disease cohort. In summary, the disease-specific key crosstalk genes are as follows (Table 5):

**Table 5:** List of key crosstalk genes specific to each disease

Disease	Specific Key Crosstalk Genes
AD	*LILRB2, TLN1, ITGAM, C5AR1, RPL15, FOS, EIF4A2, NKTR, TPT1, RBM25, RPL22, TAX1BP1, RPS6KB1, NCF4, MYO1F, VAV1, HCLS1*
ALS	*LILRB2, TLN1, ITGAM, C5AR1, RPL15, FOS, EIF4A2, NKTR, TPT1, RBM25, RPL22, TAX1BP1, RPS6KB1, IL18, PTPRC, PTEN, CTSS, TNFSF13B, TLR4, CSF1R*
PD	*LILRB2, TLN1, ITGAM, C5AR1, IL18, PTPRC, PTEN, CTSS, TNFSF13B, TLR4, CSF1R, NCF4, MYO1F, VAV1, HCLS1*

### Analysis of LASSO Model

To assess the classification capacity to distinguishing between diseased and healthy individuals, and to evaluate the importance of genes within each disease, we developed a LASSO model with cross-validation. One of the primary advantages of the LASSO model is its capacity to identify and select features (in this context, genes) that are significantly associated with the disease. Model performance was evaluated using the metrics presented in Table 6.

**Table 6:** LASSO Model evaluation metrics for each disease in original cohorts

Metrics	LASSO Model of AD	LASSO Model of ALS	LASSO Model of PD
Accuracy	0.59	0.78	0.60
Precision	0.54	0.63	0.58
Recall	0.62	0.77	0.56
Specificity	0.56	0.79	0.64
F1 Score	0.57	0.69	0.57

**Fig. 5:**
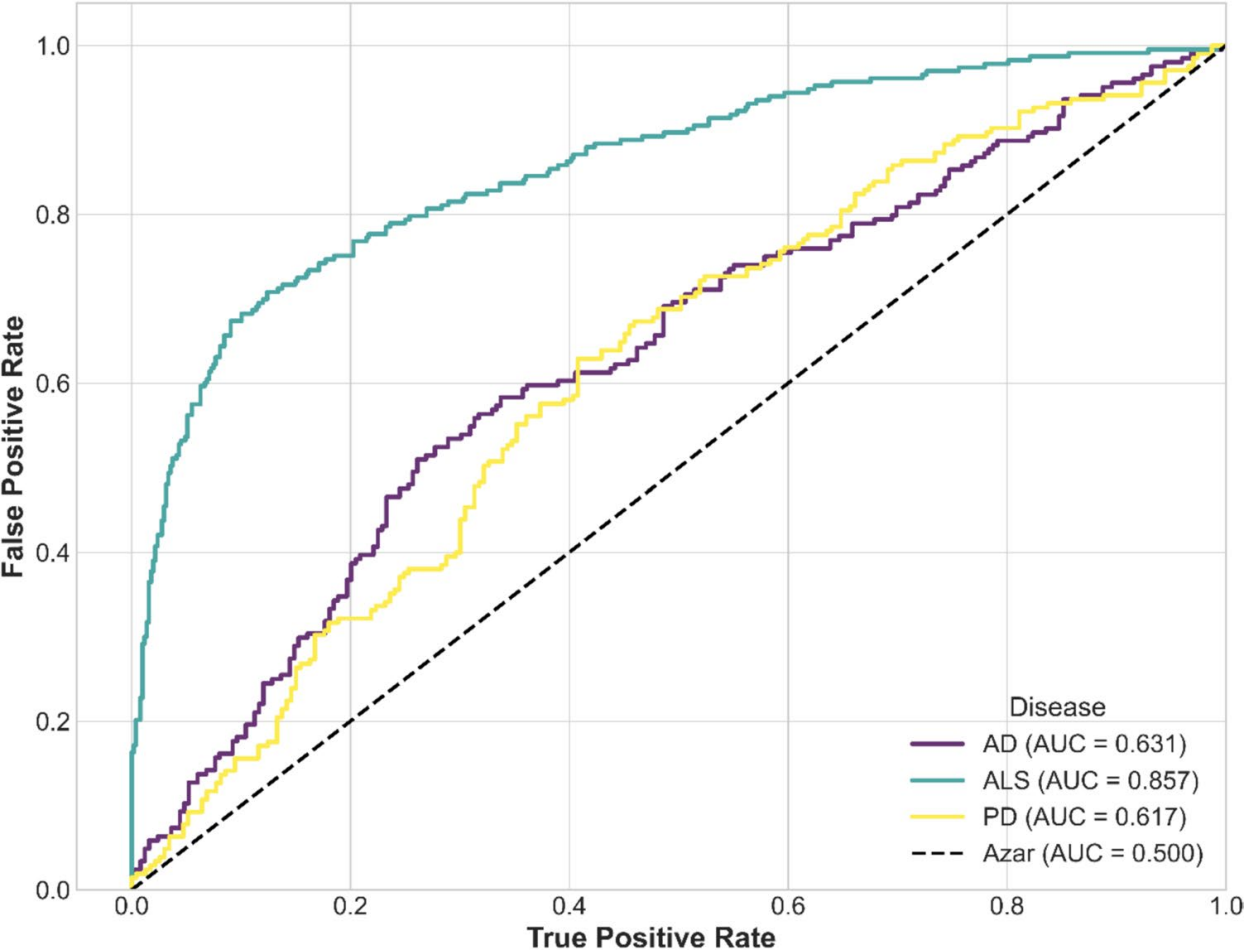
ROC Curves of the LASSO models for each of the diseases: Alzheimer’s disease (AD), Amyotrophic Lateral Sclerosis (ALS), and Parkinson’s disease (PD)

The LASSO model for ALS demonstrated the best overall performance, achieving an accuracy of 0.78, a recall of 0.77, a specificity of 0.79, a precision of 0.63, and an F1 score of 0.69. In contrast, the model for AD obtained an accuracy of 0.59, a recall of 0.62, a specificity of 0.56, a precision of 0.54, and an F1 score of 0.57. Finally, for PD, the model exhibited an accuracy of 0.60, a recall of 0.56, a specificity of 0.64, a precision of 0.58, and an F1 score of 0.57. On the other hand, the ROC curve values (Fig. 5) were 0.86 for ALS, 0.63 for AD and finally for PD an ROC value of 0.62 was obtained. Figure 2 of the Supplementary Material shows the Confusion Matrix obtained for each disease.

The model performs gene selection where a non-zero coefficient indicates that the gene has been retained and contributes to the model’s classification. On the other hand, any gene with a coefficient equal to zero was excluded from this analysis. Table 7 presents the coefficients of the selected genes for each disease.

**Table 7:** Gene coefficients estimated by the LASSO model for each of the diseases. NaN (Not applicable)

Gene	AD	ALS	PD
	Coefficients	Coefficients	Coefficients
LILRB2	−0.0127	0.2189	0.0858
TLN1	−0.1718	0.6175	0
ITGAM	0.1128	0	0
C5AR1	−0.2974	0.8024	0.1328
RPL15	0.4417	0	NaN
FOS	0.3625	−0.2093	NaN
EIF4A2	−0.1190	0.1532	NaN
NKTR	−0.2771	0.1632	NaN
TPT1	−0.0944	0.2586	NaN
RBM25	−0.2425	0.011	NaN
RPL22	−0.5097	0.2853	NaN
TAX1BP1	−0.6115	−0.2883	NaN
RPS6KB1	0.7001	0.6709	NaN
IL18	NaN	−0.5607	0.0873
PTPRC	NaN	−0.0186	−0.1115
PTEN	NaN	−0.6708	−0.0065
CTSS	NaN	0.8534	0.0425
TNFSF13B	NaN	0.4048	0
TLR4	NaN	0	0
CSF1R	NaN	−0.8222	0.1578
NCF4	0.0988	NaN	0.1677
MYO1F	−0.2452	NaN	0.0176
VAV1	0.3686	NaN	0
HCLS1	0.3984	NaN	0

The results obtained indicate the genes selected by the LASSO model, retaining only those with a coefficient other than zero and excluding all genes assigned a zero coefficient. 17 genes with non-zero coefficients were obtained in ALS, 13 genes in AD and 9 genes in PD. The ALS model demonstrated the most robust performance, highlighting the importance of genes such as *CTSS*, *C5AR1*, and *CSF1R* with particularly high coefficients. In contrast, the models for AD and PD exhibited more modest predictive performance, suggesting a lesser influence of the examined genes in their prediction. However, in AD, *TAX1BP1* and *RPS6KB1* were identified as genes with coefficients of interest.

Upon investigating genes common to all three diseases, *C5AR1* and *LILRB2* were identified. The magnitude of the absolute coefficients indicated that *C5AR1* is especially relevant in ALS (0.8024), while its influence is lower in AD (−0.2974) and PD (0.1328). *LILRB2*, on the other hand, showed limited influence across all diseases. These findings suggest a strong discriminatory capacity of these genes in blood for ALS, but inferior capacity in AD and PD, in line with the primary pathology in the brain in these two diseases.

The analysis of genes shared between ALS and AD (*C5AR1, LILRB2, TLN1, FOS, EIF4A2, NKTR, TPT1, RBM25, RPL22, TAX1BP1* and *RPS6KB1*) revealed the highest relevance for *RPS6KB1* (0.7001 in AD, 0.6709 in ALS), *TLN1* (0.6175 in ALS, −0.1718 in AD), and *TAX1BP1* (−0.6115 in AD, −0.2883 in ALS). On the other hand, the genes common to AD and PD were *C5AR1, LILRB2, NCF4* and *MYO1F*, with generally less prominent coefficients. Notably, ALS and PD share the following genes: *C5AR1, LILRB2, IL18, PTPRC, PTEN, CTSS* and *CSF1R*. It’s notable that in PD, the coefficients for these genes were low.

### Validation of the LASSO Model in Independent External Cohorts and Comparison With Other Classification Models

The validation of our findings in an independent external cohort was the final step to ensure the model’s reliability. To this end, we implemented a three-pronged validation approach to address three critical questions. First, we evaluated the generalizability of performance by quantifying whether the model trained on the initial cohort preserved its discriminatory power on the independent dataset. Second, we assessed the consistency of feature selection by retraining the LASSO model on the external cohort to determine the degree of overlap between the gene signatures identified in both analyses. Finally, given that the ALS signature was the most robust, we performed a comparative analysis to determine if advanced non-linear models could offer superior predictive performance compared with our linear LASSO model.

#### Performance of the Original Model on the Independent External Cohort

**Fig. 6:**
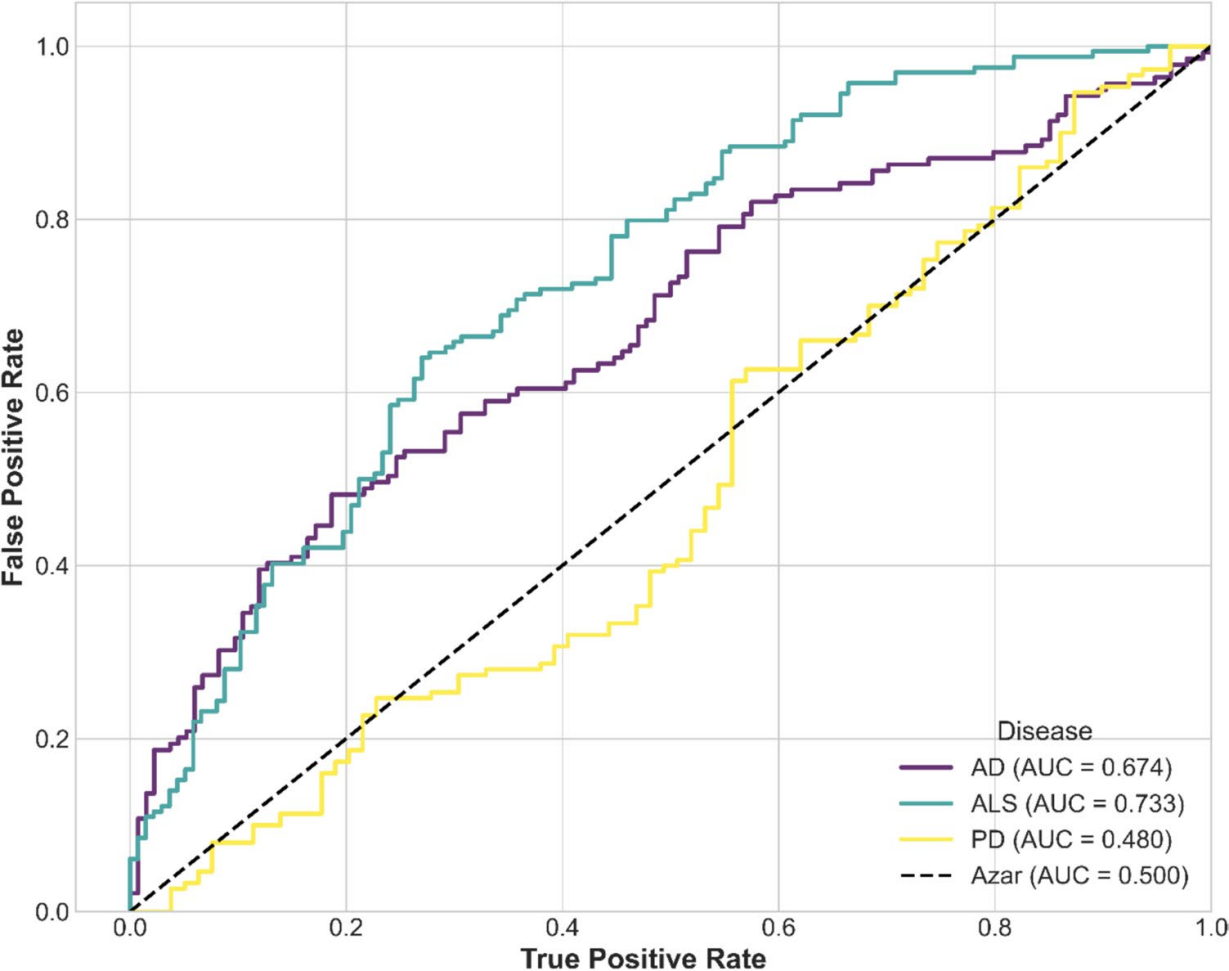
ROC Curves of the LASSO models for each of the diseases in independent external cohorts: Alzheimer’s disease (AD), Amyotrophic Lateral Sclerosis (ALS), and Parkinson’s disease (PD)

The performance of the models on the independent external cohort is shown in Table 8 and Fig. 6. The confusion matrices are presented in Fig. 3 of the Supplementary Material. The evaluation of model performance in the external cohort revealed different generalization dynamics for each disease. The ALS model, while remaining the most robust overall, experienced a decrease in its overall performance, with the AUC dropping from 0.86 in the original cohort to 0.74 in the validation set. This decline was primarily driven by a reduction in sensitivity (Recall from 0.77 to 0.46), although this came with a trade-off of increased precision (Precision from 0.63 to 0.73). In contrast, the AD model showed a slight improvement in its overall performance (AUC from 0.63 to 0.68), largely due to a substantial improvement in specificity (Specificity from 0.56 to 0.85), though it also suffered a reduction in its sensitivity (Recall from 0.62 to 0.40). Finally, the PD model demonstrated limited generalizability, with predictive performance dropping to chance levels on the new cohort. A specificity of 0.0 and a sensitivity of 1.0 indicate that the classifier predicted all samples as positive, losing all ability to identify control cases. The AUC value of 0.48, which is below chance level, confirmed the poor performance of the model.

**Table 8:** LASSO Model evaluation metrics for each disease in independent external cohorts

Metrics	LASSO Model of AD	LASSO Model of ALS	LASSO Model of PD
Accuracy	0.62	0.61	0.66
Precision	0.74	0.73	0.66
Recall	0.40	0.46	1.00
Specificity	0.85	0.80	0
F1 Score	0.52	0.56	0.79

#### Stability of the Gene Signature in the Validation Cohort

To evaluate the stability of the gene signature, the LASSO models were retrained directly on the independent external cohort. The results are shown in Table 9. For ALS, the retrained model selected 17 genes with non-zero coefficients, the same number as in the original model. Although the number of selected genes (17) was identical, the composition of the gene signature varied between the models. Specifically, the genes *TLN1, C5AR1*, and *EIF4A2*, which were present in the original model, were excluded in the retrained model. In their place, the new model selected *ITGAM, RPL15*, and *TLR4*, which were not part of the original signature. Among them, *TAX1BP1*, *CTSS* and *RPS6KB1* emerged as the coefficients with the greatest weight. In contrast, the model for AD experienced a notable decrease, selecting only four genes: *ITGAM, NKTR, RPL22* and *TAX1BP1*. A functional enrichment analysis (DAVID) of the gene signatures revealed that the AD genes are related to the immune response, while the ALS genes were associated with signal transduction and the inflammatory response (Table 6 of Supplementary Material).

**Table 9:** Gene coefficients estimated by the LASSO model for each of the diseases in independent external cohorts. NaN (Not applicable)

Gene	AD	ALS	PD
	Coefficients	Coefficients	Coefficients
LILRB2	0	−0.3364	0
TLN1	0	0	0
ITGAM	0.4712	0.3046	0
C5AR1	0	0	0
RPL15	0	0.0722	NaN
FOS	0	−0.1707	NaN
EIF4A2	0	0	NaN
NKTR	0.3225	−0.1094	NaN
TPT1	0	0.4040	NaN
RBM25	0	−0.5921	NaN
RPL22	−0.2901	0.2738	NaN
TAX1BP1	−0.2311	1.1268	NaN
RPS6KB1	0	−0.7199	NaN
IL18	NaN	−0.6752	0
PTPRC	NaN	−0.1793	0.1627
PTEN	NaN	−0.5364	0
CTSS	NaN	0.8630	0
TNFSF13B	NaN	0.5568	0
TLR4	NaN	0.2703	0
CSF1R	NaN	−0.4320	0
NCF4	0	NaN	0
MYO1F	0	NaN	0
VAV1	0	NaN	0
HCLS1	0	NaN	0

Lastly, the PD signature was reduced to a single gene, *PTPRC,* with a modest coefficient, confirming the lack of a stable predictive signal in this cohort, although it is notable that this gene has been proposed in the literature as a relevant prognostic biomarker for PD (Bottero et al., 2018).

The comparative analysis between the gene signatures from the original and the independent external cohorts reveals key insights into model stability. The ALS signature demonstrated considerable robustness in its composition, with key genes such as *CTSS*, *PTEN*, *IL18*, *TNFSF13B*, and *CSF1R* being selected in both models. *CTSS* emerged as a highly consistent feature, maintaining a high positive coefficient in both signatures (Original: 0.8534 vs. Validation: 0.8630), which reinforces its central role as a marker associated with microglia and immune system regulation (Zhang et al., 2021). Similarly, *PTEN* and *IL18* maintain a consistent negative role in both the original and validation models (*PTEN*: −0.6708 vs. −0.5364; *IL18*: −0.5607 vs. −0.6752). This suggests that the dysregulation of neuronal cell death pathways (associated with *PTEN*) and the inflammasome (associated with *IL18*), respectively, are influential and stable features of the pathology (Gasparoto et al., 2014; Wang et al., 2020). Finally, *TNFSF13B* and *CSF1R* also retain their sign, albeit with variations in coefficient magnitude, highlighting the relevance of immune mediation and microglial survival (Consiglio et al., 2022; Munro et al., 2024). However, this stability coexists with a significant readjustment in the role of other genes. Notably, *TAX1BP1* shifted from a negative coefficient (−0.2883) to become the most influential positive predictor (1.1268), and *RPS6KB1* also inverted its sign. This variation suggests that the role of these genes is highly context-dependent, possibly due to interactions with other genetic or clinical factors.

For AD, the retraining process on the validation cohort acted as a refinement filter. Unlike the original model, which identified a broad set of 13 genes, the retrained model converged on a four-gene signature (*ITGAM, NKTR, RPL22*, and *TAX1BP1*). This core signature is led by *ITGAM* (0.4712), a central gene in microglial function (Pichet Binette et al., 2024). The other genes in the signature, *NKTR, RPL22*, and *TAX1BP1*, are involved in processes such as NK cell lytic function, hematopoiesis and cellular senescence, and autophagy, respectively (Bai et al., 2022; Eubanks et al., 2024; Li et al., 2024).

Retraining of the models also revealed a molecular overlap between ALS and AD, with all four genes from this signature (*ITGAM, NKTR, RPL22*, and *TAX1BP1*) being selected in both models, pointing to the involvement of shared biological pathways. However, the most relevant finding is the differential regulation of these genes. With the exception of *ITGAM*, which maintains a positive coefficient in both pathologies, the other three genes exhibit opposing predictive roles. *TAX1BP1* and *RPL22* act as positive predictors in ALS but negative in AD, while *NKTR* shows the reverse pattern. This duality suggests that while the same pathways are perturbed in both diseases, the direction of that dysregulation is pathology-specific.

Similarly, this inverse dynamic extends to ALS and PD, where their only shared gene, *PTPRC*, exhibited a positive coefficient in PD (0.1627) but a negative one in ALS (−0.1793).

#### Comparison of the LASSO Model With Other Classification Models

To confirm that the linear LASSO model was the most appropriate choice for our ALS signature, we conducted a comparative benchmark analysis. The optimized LASSO model was compared against two robust non-linear models: SVM with RBF and RF. The models were evaluated on both the original cohort and the independent validation cohort. The performance metrics are summarized in Table 10. The graphs corresponding to the ROC curves and Confusion Matrix are presented in Figs. 5 and 6 of the Supplementary Material.

**Table 10:** LASSO, SVM and RF evaluation metrics for ALS in original cohort and independent external cohorts

Model	Cohort	AUC	Accuracy	Precision	Recall	Specificity	F1 Score
LASSO	Original	0.86	0.78	0.63	0.77	0.79	0.69
	Validation	0.74	0.61	0.73	0.46	0.80	0.56
SVM	Original	0.88	0.83	0.72	0.76	0.86	0.73
	Validation	0.57	0.53	0.61	0.38	0.70	0.47
RF	Original	0.86	0.87	0.79	0.65	0.92	0.71
	Validation	0.63	0.51	0.83	0.12	0.97	0.21

The results demonstrated the superior robustness of the LASSO model. While the nonlinear models achieved comparable or higher performance on the original cohort (SVM AUC 0.88; RF AUC 0.86), their performance decreased substantially upon validation. In the case of SVM model, the AUC dropped to 0.57. The Random Forest model’s performance fell to an AUC of 0.63, exhibiting a pronounced decrease in sensitivity (Recall = 0.12). This model almost exclusively predicted the ‘Control’ class, achieving a near-perfect Specificity of 0.97 at the cost of failing to identify 88% of ALS patients. In contrast, the LASSO model (Original AUC 0.86) proved the most stable, retaining a robust AUC of 0.74 in the validation set.

This result suggests that while the non-linear models overfitted to the training data by capturing cohort-specific variance, the simpler, linear LASSO model captured the most generalizable biological signal. This finding strongly validates the choice of LASSO as the most reliable classification method for this ALS signature. Nevertheless, it is important to note that the LASSO model also showed a reduction in performance during external validation, specifically a decrease in accuracy of approximately 18% and in recall of ~ 30%. This decline indicates a degree of overfitting to the original training data. As documented in similar machine learning studies, these reductions are primarily attributed to the inherent biological heterogeneity of high-dimensional whole-blood transcriptomic data (Huang et al., 2025; Yap et al., 2025).

## Discussion

To the best of our knowledge, this study presents the first comparative analysis of the systemic transcriptomic background in three major neurodegenerative diseases (ALS, AD, and PD) using both supervised and unsupervised approaches. The objective of this study was to leverage the shared pathogenic basis in an accessible tissue, such as whole blood, to develop systemic biomarkers (Henriksen et al., 2014), thereby addressing an urgent clinical need. Our investigation tackled this challenge using a rigorous LASSO-based design, which simultaneously selected the most influential predictors and evaluated their classification power.

Our results underscore a critical divergence in the viability of these biomarkers: while ALS presented a robust genetic signature in blood, validated across two independent cohorts (AUC ≥ 0.73), the models for AD and PD displayed a much more subtle signal. Although the AD model showed a slight improvement upon validation (AUC from 0.63 to 0.68), its refined core of four genes (*ITGAM, NKTR, RPL22, TAX1BP1*) remained significantly less predictive than the ALS model. This was further evidenced by the limited predictive capacity of the PD model. This attenuated signal in AD and PD suggests that, unlike ALS, the primary pathology of these diseases may be predominantly confined to the CNS, resulting in a minimal systemic transcriptomic footprint. This feature is well-documented in AD. While recent research has found success with plasma protein biomarkers (such as p-tau), the search for a robust and replicable transcriptomic signature for AD in blood has been largely unsuccessful (Shvetcov et al., 2023). The core CNS pathology of AD (amyloid plaques and tau tangles) appears to have a weak peripheral transcriptomic reflection, which is easily masked by systemic confounders like aging and low-grade inflammation. Conversely, ALS is increasingly recognized not just as a motor neuron disease, but as a multisystemic disorder involving significant peripheral immune and metabolic dysregulation (Hong et al., 2025). It is likely this strong, systemic inflammatory signature, reflected in the blood, explains why our ALS models substantially outperformed those for AD and PD.

This CNS confinement is particularly evident in PD. The PD model showed low performance (AUC 0.62) and did not improve in the validation cohort (AUC 0.48), retaining only a single gene (*PTPRC*). This decline could be due to a combination of technical and biological factors. First, the scarcity of large blood cohorts for PD required us to construct a validation cohort by integrating three small and technically disparate datasets. Despite applying ComBat batch correction, it is highly probable that the residual technical noise from this integration masked an already weak biological signal. Second, unlike the strong systemic inflammatory signature observed in ALS, PD pathology is predominantly confined to the CNS. This suggests that transcriptomic changes in peripheral blood may be too inconsistent to serve as a robust biomarker. Actually, the search for reliable blood biomarkers for PD remains one of the biggest challenges in this field because they lack the necessary specificity and sensitivity in this tissue (Liu et al., 2024). Therefore, the collapse of the PD model likely reflects both the technical limitation of our validation cohort and an intrinsically weak systemic signal for this pathology.

The robustness of the ALS model is the central finding of this study. This robustness is not only demonstrated by its stability in the external validation cohort, but also further confirmed by a comparative analysis (see Table 10), which showed that complex non-linear models (SVM and Random Forest) overfitted the training data and exhibited limited generalizability. Although the LASSO model proved superior, it also experienced a reduction in performance during external validation, as detailed in the Results section. This decline reflects a degree of overfitting that can be attributed to the use of whole-blood transcriptomic data, which, while easily accessible and minimally invasive, is inherently heterogeneous and highly dimensional. The substantial biological heterogeneity across independent cohorts frequently causes such generalization bottlenecks. Indeed, similar machine learning applications in blood transcriptomics for other complex conditions consistently report drastic reductions in performance metrics upon external validation (Huang et al., 2025; Yap et al., 2025). Furthermore, this study focused solely on gene expression data, which, although informative, does not capture the full complexity of the disease’s pathogenesis. The integration of additional omics data, such as proteomics, metabolomics, and epigenetics, could provide a more comprehensive understanding of the underlying molecular mechanisms and improve the predictive power of the models. Nevertheless, the LASSO model’s ability to maintain an AUC of 0.74 is highly significant, indicating that it successfully captured a resilient biological signal despite the study’s limitations. Future research should focus on addressing these challenges and validating the findings in larger independent cohorts to strengthen the evidence base for the identified biomarkers.

It is crucial to emphasize that the ALS model’s strength does not rely on a single proinflammatory marker. Its predictive power and stability across both cohorts stem from the LASSO’s ability to integrate a complex, multifaceted signature of immune dysregulation. The true ALS signature captured in blood is not simply inflammation, but a composite state that simultaneously reflects microglial activation (*CTSS*), systemic immune depletion (*IL18, PTPRC, CSF1R*), and CNS glial dysfunction (*PTEN*). We contend that a model based solely on simple inflammatory markers would have been insufficient. The novelty of our finding is that this nuanced, multi-pillar signature provides robust and stable disease classification.

At the core of this signature is the *CTSS* gene (Cathepsin S), which was consistently selected in both cohorts as the positive predictor with the highest coefficient. This finding validates the hypothesis that neuroinflammation is not a secondary event, but a central, detectable signal of ALS pathology. *CTSS* is a protease preferentially expressed by microglia, macrophages, and dendritic cells to regulate antigen presentation. Our results, which associate high *CTSS* expression in blood with ALS diagnosis, are consistent with the biological evidence of its overexpression in the spinal cord of ALS patients and its established role in microglia-mediated neurotoxicity (Berjaoui et al., 2015; Liu et al., 2025; Nakanishi, 2020; Tang et al., 2024; Zhang et al., 2021, 2025). Therefore, the selection of *CTSS* by our LASSO model reflects a critical biological signal, demonstrating that microglial activation, an event previously thought to be confined to the CNS, generates a systemic footprint detectable in the blood.

This signal was reinforced by *TNFSF13B* (*BAFF*). Its selection supports the hypothesis of immune dysregulation, yet its role is complex. While deficiency of its receptor (*BAFF-R*) accelerates ALS in animal models (Consiglio et al., 2022; Stelmach et al., 2020; Tada et al., 2013), our results associate elevated systemic levels with the pathology. We suggest that these high levels do not reflect a protective role, but rather act as an indicator of sustained immune cell activation (innate and B cells) that contributes to the neurotoxic environment.

While *CTSS* represents the immune activation signal, the model’s stability is equally supported by negative predictors that reveal the dysfunctional side of this pathology. *PTEN* is a clear example of glial dysfunction. Our model associates lower systemic *PTEN* levels with the disease, a biologically complex finding. Although *PTEN* inhibition can be neuroprotective for motor neurons (Wang et al., 2021), its loss-of-function in glia promotes reactive astrogliosis and hinders myelination (Zhang et al., 2019; Zheng et al., 2025), both of which are central neurotoxic processes in ALS. It should be noted that *PTEN* production in motor neurons is primarily local and may not correlate directly with whole blood levels. Our hypothesis is that the *PTEN* signal detected in the blood does not reflect a neuronal compensatory mechanism, but rather is a reflection of this glial dysfunction within the CNS.

This systemic footprint of dysfunction is complemented by *IL18*. Although a potent proinflammatory cytokine produced by the NLRP3 inflammasome (which is associated with CNS neurotoxicity) (Begum et al., 2025; Chen et al., 2023; Heavener et al., 2025; Ren et al., 2024; Xu et al., 2025), our results show lower blood levels associated with ALS. This suggests a disconnection between central inflammation and the systemic response: while the NLRP3 inflammasome remains hyperactive in the microglia, the peripheral immune system measured in the blood may enter a state of immune exhaustion (Schwartz & Colaiuta, 2024). While this hypothesis requires functional validation, we speculate that these low levels reflect the progression of the chronic disease.

*PTPRC*, a crucial regulator of autoantibody overproduction by B lymphocytes (Huang et al., 2024; Torres Iglesias et al., 2024), was also identified as a stable negative predictor. Scientific evidence linking this gene to ALS is sparse, save for one study in an ALS model (Drosophila) suggesting that *PTPRC* suppression may reverse neurodegeneration (Pun et al. 2022). Regardless of the limited translational value of that finding, our results (low systemic levels associated with the disease) are interpreted not as a protective response, but within the context of chronic pathology. We propose that, similar to *IL18*, these low levels reflect an exhaustion of the lymphoid lineage. In the setting of chronic ALS and immunosenescence, low *PTPRC* blood levels would not represent a beneficial suppression, but rather act as a biomarker of this systemic immune dysfunction and disease progression.

Finally, the complexity of the immune signal in ALS is encapsulated in *CSF1R*. This gene, crucial for microglial and monocyte survival (Chen et al., 2024), was identified as a stable negative predictor. This finding is notable given that *CSF1R* inhibition is a neuroprotective therapeutic strategy in ALS models (Han et al., 2022). Our hypothesis is that low blood levels of *CSF1R* would reflect this peripheral immune exhaustion—an indicator of myeloid lineage dysfunction in the context of chronic disease.

To conclude, the comparison with the AD signature reinforces the specificity of our ALS model. The refined four-gene core for AD (*ITGAM, NKTR, RPL22, TAX1BP1*) was entirely contained within the ALS signature, confirming a shared pathogenic background centered on the immune response. However, this common background manifests through marked differential regulation.

*RPL22* emerges as the most robust finding from this comparison. It was the only gene selected in both cohorts for both diseases that maintained a stable coefficient with an opposite sign (positive in ALS; negative in AD). This demonstrates that, while the same pathways are implicated, the direction of their dysregulation is pathology-specific. *RPL22* is a ribosomal protein involved in T-cell development and the regulation of endoplasmic reticulum stress signal transduction (Chen et al., 2025; Su et al., 2023). Although there is little literature directly linking it to ALS or AD, its known biological function is highly relevant. *RPL22* has recently been shown to be an active promoter of cellular senescence, a key process in aging (Li et al., 2024). Our results show elevated systemic *RPL22* levels in ALS patients, which could reflect a state of accelerated cellular senescence. This state may contribute to a chronic pro-inflammatory environment. This finding is particularly relevant when contrasted with AD, where *RPL22* is decreased, demonstrating that ALS and AD, while both age-related diseases, possess fundamentally distinct systemic senescence signatures. In contrast to the stability of *RPL22*, the absence (*ITGAM*) or instability of other shared genes (*TAX1BP1, NKTR*), which inverted their predictive roles even within the same disease, underscores that their function is highly dependent on the clinical or genetic context of each cohort.

Similarly, the pathological relationship between ALS and PD is reflected by the *PTPRC* gene. This gene was the only feature retained in the PD model (with a positive coefficient), whereas it acted as a negative predictor in ALS. This inverse relationship mirrors the dynamic observed with *RPL22* in AD, further reinforcing the concept that while these neurodegenerative diseases share critical nodes of immune vulnerability, the systemic direction of this immune dysregulation is disease-specific.

In summary, this comparative transcriptomic approach suggests that the peripheral signature of ALS is characterized by a broader and more robust immune-related signal, whereas AD and PD display more restricted or localized transcriptomic alterations. Despite sharing certain molecular intersections with ALS, these diseases appear to differ in the directionality of their regulatory patterns, potentially reflecting distinct systemic responses to neurodegeneration.

## Conclusion

From a translational perspective, this study has identified a specific transcriptomic signature and developed a robust classification model for ALS providing proof-of-concept that systemic blood-based gene expression can support disease classification. The primary advantage of this approach lies in its clinical applicability, as this RNA signature could be quantified from whole blood samples using standard technologies such as RT-qPCR. Future studies in larger, multicenter, and, crucially, longitudinal cohorts are needed to determine not only its diagnostic utility but also its prognostic and progression-monitoring potential. Moreover, future mechanistic studies will be important to further elucidate the biological link between the systemic expression of these genes and the core pathology in the CNS. In conclusion, this study not only delineates a distinct systemic immune-transcriptomic pattern in ALS, separate from other neurodegenerative diseases, but also establishes interpretability and linear simplicity as essential factors for developing reproducible blood-based biomarkers with clinical translational potential.

## Supplementary Information

Below is the link to the electronic supplementary material.

Supplementary file1 (XLSX 2920 KB)

## Data Availability

The datasets analysed during the current study are available in the Gene Expression Omnibus (GEO) repository (https://www.ncbi.nlm.nih.gov/geo/). Specifically, the discovery cohorts correspond to accession numbers GSE112676 (https://www.ncbi.nlm.nih.gov/geo/query/acc.cgi?acc=GSE112676), GSE99039 (https://www.ncbi.nlm.nih.gov/geo/query/acc.cgi?acc=GSE99039), and GSE140829 (https://www.ncbi.nlm.nih.gov/geo/query/acc.cgi?acc=GSE140829). The independent validation cohorts are available under accession numbers GSE112680 (https://www.ncbi.nlm.nih.gov/geo/query/acc.cgi?acc=GSE112680), GSE57475 (https://www.ncbi.nlm.nih.gov/geo/query/acc.cgi?acc=GSE57475), GSE72267 (https://www.ncbi.nlm.nih.gov/geo/query/acc.cgi?acc=GSE72267), GSE18838 (https://www.ncbi.nlm.nih.gov/geo/query/acc.cgi?acc=GSE18838), and GSE63061 (https://www.ncbi.nlm.nih.gov/geo/query/acc.cgi?acc=GSE63061). All data used in this research are publicly accessible and were processed as described in the Materials and Methods section. All source code is available in the GitHub repository (https://github.com/egasconr/SourceCode-Paper-3).

## Glossary

AD: Alzheimer’s Disease
ALS: Amyotrophic Lateral Sclerosis
ARG1: Arginase 1
AUC: Area Under the Curve
BIRC3: Baculoviral IAP Repeat Containing 3
C5AR1: Complement C5a Receptor 1
CNS: Central Nervous System
CORO1A: Coronin 1A
CSF1R: Colony Stimulating Factor 1 Receptor
CSF3R: Colony Stimulating Factor 3 Receptor
CTSS: Cathepsin S
DAVID: Database for Annotation, Visualization and Integrated Discovery
DEGs: Differentially Expressed Genes
EIF4A2: Eukaryotic Translation Initiation Factor 4A2
ERBB2: Erb-B2 Receptor Tyrosine Kinase 2
FC: Fold Change
FDR: False Discovery Rate
FGR: FGR Proto-Oncogene, Src Family Tyrosine Kinase
FN: False Negative
FOS: Fos Proto-Oncogene, AP-1 Transcription Factor Subunit
FP: False Positive
GEO: Gene Expression Omnibus
GNLY: Granulysin
GO: Gene Ontology
GZMB: Granzyme B
HCK: HCK Proto-Oncogene, Src Family Tyrosine Kinase
HCLS1: Hematopoietic Cell-Specific Lyn Substrate 1
IL18: Interleukin 18
IRF4: Interferon Regulatory Factor 4
ITGAM: Integrin Subunit Alpha M
LASSO: Least Absolute Shrinkage and Selection Operator
LILRB1: Leukocyte Immunoglobulin Like Receptor B1
LILRB2: Leukocyte Immunoglobulin Like Receptor B2
MAPK1: Mitogen-Activated Protein Kinase 1
MMP9: Matrix Metallopeptidase 9
MSN: Moesin
MYO1F: Myosin IF
NCF1: Neutrophil Cytosolic Factor 1
NCF4: Neutrophil Cytosolic Factor 4
NKTR: Natural Killer Cell Triggering Receptor
OPTN: Optineurin
PD: Parkinson’s Disease
PFN1: Profilin 1
PGLYRP1: Peptidoglycan Recognition Protein 1
PRF1: Perforin 1
PTEN: Phosphatase and Tensin Homolog
PTPN6: Protein Tyrosine Phosphatase Non-Receptor Type 6
PTPRC: Protein Tyrosine Phosphatase Receptor Type C
RBF: Radial Basis Function
RBM25: RNA Binding Motif Protein 25
RF: Random Forest
RGS19: Regulator of G-Protein Signaling 19
ROC: Receiver Operating Characteristic
RPL15: Ribosomal Protein L15
RPL22: Ribosomal Protein L22
RPS29: Ribosomal Protein S29
RPS6KB1: Ribosomal Protein S6 Kinase B1
SPI1: Spi-1 Proto-Oncogene
SVM: Support Vector Machine
TAX1BP1: Tax1 Binding Protein 1
TLN1: Talin 1
TLR4: Toll Like Receptor 4
TN: True Negative
TNFSF13B: TNF Superfamily Member 13b
TP: True Positive
TPT1: Tumor Protein, Translationally-Controlled 1
URI1: URI1 Prefoldin Like Chaperone
VAV1: Vav Guanine Nucleotide Exchange Factor 1
WAS: Wiskott-Aldrich Syndrome
WGCNA: Weighted Gene Co-expression Network Analysis
ZRANB2: Zinc Finger RANBP2-Type Containing 2
